# Understanding the Parental Caregiving of Children with Cerebral Palsy in Saudi Arabia: Discovering the Untold Story

**DOI:** 10.3390/ijerph22060946

**Published:** 2025-06-17

**Authors:** Ashwaq Alqahtani, Ahmad Sahely, Heather M. Aldersey, Marcia Finlayson, Danielle Macdonald, Afolasade Fakolade

**Affiliations:** 1 Department of Physical Therapy, College of Applied Medical Sciences, Qassim University, Almulaida 52571, Saudi Arabia; 2 School of Rehabilitation Therapy, Queen’s University, Kingston, ON K7L 3N6, Canada; hma@queensu.ca (H.M.A.); marcia.finlayson@queensu.ca (M.F.); a.fakolade@queensu.ca (A.F.); 3 Department of Physical Therapy, College of Nursing and Health Sciences, Jazan University, Jazan 45142, Saudi Arabia; asahely@jazanu.edu.sa; 4 School of Nursing, Queen’s Health Sciences, Queen’s University, Kingston, ON K7L 3N6, Canada; danielle.macdonald@queensu.ca; 5 Providence Care Hospital, Kingston, ON K7L4X3, Canada

**Keywords:** cerebral palsy, child, navigating care child, experiences, needs, parental caregiving, Saudi Arabia, social ecological, culture, support systems

## Abstract

Parents provide most of the support needed for children with cerebral palsy (CP) to increase the child’s participation and independence. Understanding the experiences of parents caring for children with CP is essential for developing effective family programs and services. The current knowledge about parents’ experiences in CP is based on studies in Western countries, with little known about this phenomenon in Arab countries like Saudi Arabia. This study aimed to understand the unique experiences and support needs of Saudi parents caring for children with CP from a social-ecological perspective. We conducted a qualitative, exploratory, descriptive study involving 12 semi-structured interviews with mothers and fathers of children with different types of CP. We analyzed the data using a reflexive thematic approach, following six distinct phases. Participants’ narratives revealed a complex caregiving journey marked by both challenges and rewards. Support from Saudi nuclear and extended family members was considered important; however, many parents expressed a need for additional physical and financial assistance from their families. Parents reported feeling stressed and experiencing challenges in accessing and navigating educational and healthcare services. Our findings highlight that Islamic values play a crucial role in the experiences of Saudi parents. These values foster a sense of collectivism, highlighting the importance of family support and community involvement, which can affect the Saudi caregiving environment. Parents remain an essential yet often invisible part of the Saudi caregiving system. Without adequate support, parents are at risk of experiencing social, financial, academic, physical, and mental health challenges, which may affect their overall family well-being. Future work may need to consider spiritual and gender roles when developing programs or services to support Saudi parents of children with CP.

## 1. Introduction

Cerebral palsy (CP) is a leading cause of childhood physical disability, affecting over 17 million people globally [1]. In Saudi Arabia, CP prevalence ranges from 1.6 to 3.5 per 1000 live births [1,2]. Types of CP include monoplegia, which refers to the impairment of only one limb; hemiplegia, which denotes the impairment of the arm and leg on one side of the body; diplegia, referring to the involvement of the lower half of the body; and quadriplegia, which refers to the impairment of all four limbs [2,3,4]. These classifications reflect the wide range of potential impairments seen in children with CP. Children with CP may exhibit a range of difficulties, including motor, speech, visual, and intellectual impairments, which can hinder growth, limit participation, and reduce overall well-being [5]. Additionally, children with CP may experience feelings of being different, rejection, and hopelessness, which can negatively affect their self-esteem [6]. Consequently, many children with CP often need emotional, physical, and instrumental support to participate and maintain independence [7].

In most Arab families, parents (usually mothers) are the primary caregivers for children with CP [8], providing the support needed by these children. Unfortunately, caregivers of children with CP often face significant challenges and experience a range of stressors [3,9], leading to poor physical and psychological health, and reduced overall quality of life [9,10]. For example, parents of children with CP across the world have a higher prevalence of mental health conditions such as depression and anxiety compared to parents of typically developing children [11,12,13].

The literature on caregiving and support needs for parents of children with CP is growing, but current knowledge is limited in two main respects. First, current research about CP caregiving has predominantly focused on the perspectives of mothers of children with CP [14,15,16,17]. Second, most of the studies have been conducted in Western countries [18,19,20,21,22], with less attention given to the experiences of parents of children with CP in other cultural contexts. We cannot assume that the findings of studies conducted in Western countries are applicable or relevant in other cultural contexts. Indeed, our recent scoping review [8] highlighted a scarcity of information regarding the experiences and support needs of parents of children with CP in Arab contexts, including Saudi Arabia. This gap is particularly critical given the distinct cultural, familial, and religious dynamics that shape caregiving roles in the region. For instance, Islamic values, gender norms, and strong intergenerational family structures may uniquely influence the experience and support of caregiving in Saudi Arabia [23,24,25]. Furthermore, previous research conducted globally [10,26,27,28,29,30] indicates that factors influencing caregiving experiences can span individual, interpersonal, community, and societal levels.

At the individual level, attributes such as resilience, self-efficacy, and knowledge about CP can affect caregivers’ ability to manage caregiving challenges [27]. Across many cultures, interpersonal relationships within families and social networks shape how caregiving responsibilities are perceived, the availability of support systems, and overall emotional health [10]. For instance, a study on parenting success and challenges for families with children with disabilities found that strong marital relationships in Western societies improve parent–child interactions and care quality by providing emotional support and reducing stress [26]. At the community level, health and social care services, educational resources, support groups, and respite care services can significantly influence caregivers’ experiences [10,29,30]. Additionally, societal factors such as cultural norms and policies can hinder or facilitate social inclusion and access to resources among parents of children with CP [28].

The caregiving experiences of parents of children with CP in Saudi Arabia remain underexplored. Specifically, there is a lack of insight into how various multilevel individual, interpersonal, community, and societal factors influence parents’ experiences. Addressing this knowledge gap is essential for identifying the specific types of support that caregivers require to improve their ability to care for their children with CP. Hence, this study aimed to explore the caregiving experiences of Saudi parents of children with CP and characterize their support needs from a social-ecological perspective.

### Theoretical Framework

**Figure 1:**
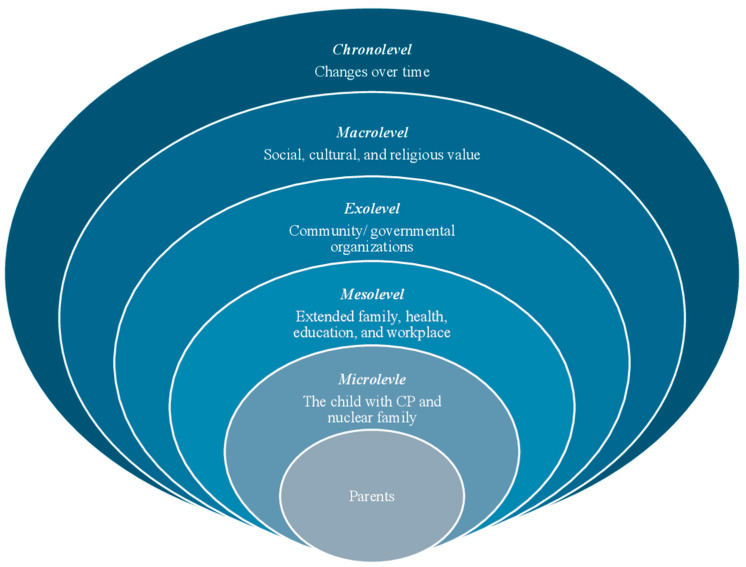
The social-ecological framework.

The social-ecological framework [31] provided a theoretical lens for this study (Figure 1). We chose this framework as it aligns with our research aim and supports methodological congruence [32]. By using the social-ecological framework to explore the experiences and needs of Saudi parents of children with CP, we aimed to develop a more comprehensive understanding of the multifaceted nature of parental caregiving experiences while acknowledging cultural and social values that shape these experiences.

The framework presents different levels of contextual influence on parents’ experiences, where each level has its own set of norms, rules, and roles depending on the setting. After identifying the personal characteristics of parents (e.g., age, gender, education), we began with the microlevel, focusing on daily caregiving routines and relationships within the nuclear family. The mesolevel examined parents’ interactions with immediate settings, such as healthcare providers and extended family networks. The exolevel addressed broader structures that indirectly affect parents, including access to community and government organizations. At the macrolevel, we explored broader cultural norms, religious beliefs, and social expectations that influence how caregiving is understood and practiced. Finally, the chronolevel captured how caregiving evolves in response to the child’s development and shifting family dynamics. This layered approach enabled a comprehensive understanding of caregiving within the Saudi cultural and societal context [14,15,16,33,34].

## 2. Materials and Methods

### 2.1. Study Design

We used an exploratory, descriptive qualitative research design [35]. This design was chosen because it allows for in-depth exploration of participants’ experiences while also providing a structured description of the phenomenon under study. Given the limited literature on parental caregiving for children with CP in Saudi Arabia, this approach enabled us to remain open to generating themes while developing findings that are both contextually grounded and relevant to practice. Researchers often use this type of research design to explore a topic with limited coverage in the literature [35], as in this study.

### 2.2. Philosophical Assumptions

This study was framed by epistemological constructivism and ontological relativism, assuming the existence of multiple, subjective, situated realities that are mind- and context-dependent [36,37]. We sought to understand the perspectives of Saudi parents of children with CP related to their caregiving experiences and support needs. Realizing that each of their experiences and realities is unique and valid and may be interpreted and described differently aligns with our philosophical assumptions.

### 2.3. Positionality Statement

The first author (AA) is a female doctoral student and Saudi pediatric physical therapist with clinical experience providing care to children with CP and their families. The second author (AS) is a male Saudi physical therapist with clinical and research experience working with persons with disabilities and their families. Our experiences as local researchers provided a comprehensive understanding of the cultural, social, and economic contexts of Saudi parents, allowing a deeper and more nuanced interpretation of participants’ experiences [38]. The other four authors (D.M., M.F., H.M.A., and A.F.) are non-Saudi female researchers with experience working with persons with disabilities and/or their family caregivers and expertise in conducting qualitative research.

### 2.4. Participant Recruitment

We used multiple strategies to recruit the participants. The first author (A.A.) contacted the Alhamah Center and the physiotherapy department at the Maternity and Children’s Hospital to obtain permission to be present during clinic hours and to request that the therapists provide her contact details to potential participants interested in the study. Also, study recruitment letters were distributed through private and public rehabilitation clinics in Qassim.

Regardless of the recruitment method, interested individuals were invited to contact the first author via phone for more information about the study. Upon contact, the first author reviewed the study information, answered any questions, obtained consent, and confirmed eligibility. Participants who indicated consent and confirmed eligibility were scheduled for an in-person/virtual interview at their convenient location, day, and time by the first author. A purposive sampling strategy was used to ensure a diverse representation of parents’ gender, children’s age, and type of CP. Participants were not compensated for their participation.

### 2.5. Eligibility Criteria

Participants could take part in the study if they met the following criteria: (1) parents of a child (0– <18 years old) with CP, (2) parents who are aged 18 years and older, (3) able to communicate in Arabic (the national language in Saudi Arabia), and (4) able and willing to arrange their own transportation to the interview location (for in-person interviews only). We excluded paid caregivers (e.g., maids, personal support workers, etc.) and parents of adults with CP.

### 2.6. Data Collection

We used a semi-structured interview guide informed by Bronfenbrenner’s social-ecological framework [31]. The first author (AA) conducted all interviews in Arabic. While 16 interviews were initially scheduled, four were canceled due to participant no-shows and non-response to rescheduling attempts. As a result, a total of 12 interviews were conducted–11 face-to-face and one via Zoom (version 6.0.11, Queen’s University, Kingston, ON, Canada). The interviews took place in a private room at the Alhamah Center and Maternity and Children’s Hospital.

Before each interview, participants received a copy of the information letter for their records and provided written informed consent. Then, participants completed a socio-demographic questionnaire that captured information about the participant’s age, sex, marital status, education level, employment status, and income level. Additionally, the questionnaire collected information about their child’s age, CP type, and motor skills. The interviews lasted between 25 and 95 min and were audio recorded. The first author transcribed the audio recordings verbatim in Arabic.

We used the information power concept to determine sufficient data (i.e., the more relevant information a sample contains, the fewer participants are needed for the actual study) [39]. According to information power elements, our study had a clearly defined aim and employed the social-ecological framework to guide data collection and analysis, which supported the generation of meaningful insights. The sample consisted of 12 purposefully selected parents who were primary caregivers of children with CP in Saudi Arabia, making the sample both specific and relevant to the study’s aim. Interviews were in-depth, conducted in the participant’s native language, and yielded rich, detailed narratives. These elements together indicated that our sample had sufficient information power to address the study’s aim, aligning with our study design that prioritizes diverse and rich accounts of a new phenomenon [35,39].

### 2.7. Data Analysis

We analyzed the data using inductive and deductive reflexive thematic analysis to ensure a comprehensive and flexible exploration of the data [40,41]. The initial analysis was carried out in Arabic by the first author (AA). The first author removed any identifying information from the transcripts and assigned codes for participant confidentiality. Transcripts were then imported into NVivo (Version 12, QSR International Pty Ltd., Melbourne, Australia) to facilitate efficient coding and data organization. In the first phase, which focused on familiarization, the first author read the transcripts multiple times to become deeply immersed in the data. In the second phase, she systematically generated codes by identifying key points of interest within the data.

In the third phase, the first author collapsed extracted codes with shared meanings into initial themes that reflected commonalities in participants’ experiences. She used a word table to sort the codes, which had been translated into English, into initial themes and to refine and adjust the themes as understanding of the data evolved. The second author, who is fluent in both Arabic and English and familiar with the subject matter, verified the accuracy of the translation of codes from Arabic to English to ensure the integrity and consistency of the data analysis. In addition, the second (A.S.) and last (A.F.) authors acted as “critical friends”, challenging initial codes/themes, contributing to new codes/themes, and stimulating reflection on alternate perspectives [42,43,44].

In the fourth phase, theme development involved revisiting the codes and compiling quotes to illustrate ideas. The first author selected exemplar quotations directly from the Arabic transcripts based on their relevance and ability to vividly illustrate the key themes. After selecting the quotations, the first author translated them into English. Once the translations were complete, the table of codes, themes, and exemplar quotations were shared with the second author. Then, all themes and quotations were shared with the remaining authors.

The fifth phase involved ongoing discussion between the first and last authors to clarify each theme’s scope, boundary, and alignment with research objectives. We developed theme names based on clear differences between ideas, using quotes from participants to maintain close connections between themes and data. In the final phase, a coherent written report was created, incorporating exemplar quotations to develop an understanding of the themes.

### 2.8. Study Quality

Grounded in our philosophical assumptions and employing a reflexive thematic analysis, we adopted multiple strategies to enhance the quality and rigor of this study. We first established the topic’s significance and relevance by focusing on understanding the experiences and support needs of Saudi parents of children with CP. This topic has the potential to positively impact the development of effective support systems that enhance parental well-being and improve outcomes for children with CP in Saudi Arabia [45]. The first author maintained a reflexive journal throughout the study, ensuring ongoing self-reflection and transparency regarding methods, challenges, and decisions [41,46,47]. To enhance credibility, we purposively included both fathers and mothers (interviewed separately), ensuring gender diversity and equal participation to provide a broader range of caregiving perspectives, prioritizing a richer interpretation of meaning through diverse perspectives [40,45]. We also engaged in “critical friend” discussions [48], which promoted reflexive dialogue and encouraged alternative interpretations of the data beyond simple consensus. We provided a thick description of participants’ experiences, including contextual details about sociocultural factors. This rich contextualization enables readers to assess the relevance of findings to other contexts [41].

To maintain methodological congruence and dependability, we consistently ensured compatibility between the study’s aim, methods, and interpretations [41,45]. Finally, all authors collaboratively reviewed and provided feedback on the findings and the written manuscript to ensure credibility and confirmability.

### 2.9. Ethical Considerations

Ethical approval was obtained from the Queen’s University Health Sciences and Affiliated Teaching Hospitals Research Board (Ref. No-6039399) and from the Saudi National Committee of Bio & Med. Ethics (Ref. No. H-04-Q-001). Written informed consent was obtained from participants who voluntarily participated in the study interviews. All participants received a copy of their signed written consent, and the researcher kept the original.

## 3. Results

### 3.1. Participant Characteristics

Analysis of socio-demographic variables showed that participants represented parents of children (ages 2–16) with different types of CP. There was an equal distribution of mothers and fathers (50% each) whose children with CP were mostly boys. The mean age of the participants was 45 ± 11 years old. Most participants were married (*n* = 11) and unemployed (*n* = 9). The characteristics of parents and their children with CP are detailed in Table 1.

### 3.2. Themes

We identified four themes from the analysis of the interview transcripts: (1) the complexity of the caring journey; (2) the value of family and external support; (3) the quality of educational and healthcare services; and (4) recommendations for community-based programming, resources, and policies. Below, we present the themes in detail, including exemplar quotations for context. Quotations are referenced by participant identifiers. For instance, F01 denotes a father, and M03 denotes a mother.

#### 3.2.1. Theme 1: The Complexity of the Caring Journey

This theme captured how parents tried to balance the weight of their caregiving responsibilities with their other daily life activities and roles. Moreover, this theme examines the positive and negative aspects of parental caregiving. Mothers and fathers differed in their descriptions of their caregiving responsibilities, with mothers sharing that they were primarily responsible for the child’s physical care, including feeding, hygiene, and providing learning support. Fathers tended to be responsible for providing transportation and meeting the family’s financial needs. Several parents shared that they often prioritized their caregiving responsibilities to the detriment of their own needs and the needs of other family members. For instance, a mother of a 3-year-old girl with diplegia CP shared:

*My husband made the heartfelt decision to sell his car to help get our daughter a wonderful new wheelchair. As a result, I’ve put my master’s studies and work on hold to focus on supporting my son and meeting his needs. It’s a big change, but I truly believe it’s the right thing to do for our family needs.*

(M05)

Some parents reported experiencing physical health issues (e.g., back and knee pain when lifting and transferring) and increased caregiver burden due to the physical and psychological demands of caregiving. When probed further about the psychological impacts, both mothers and fathers described an increased sense of burden due to an inherent belief that no one else understands their children with CP as they do and that they are the only ones capable of handling their complex care needs. For instance, the mother of a 16-year-old boy with quadriplegia CP shared this reflection:

*I regularly impose a significant emotional burden on myself concerning my son’s condition. Despite my persistent efforts to provide him with the best possible support and care, I can’t shake the feeling that I am falling short of his needs. This thought frequently occupies my mind, intensifying my awareness of the heavy responsibilities I am not doing enough for him weighs heavily on my shoulders, often leading to moments of self-doubt and anxiety about my parenting capabilities.*

(M12)

Despite their challenging caregiving contexts, several parents described persevering and maintaining hope. Parents shared many positive stories about how their caregiving provided them with knowledge and skills that continue to support their growth and development as they navigate their daily lives and roles. On many occasions, parents attributed their positive attitude and acceptance to two Islamic and cultural concepts: Tawakkul (trust and reliance on Allah) and Saber (patience), believing that having a child with CP has increased their reliance on Allah and taught them patience and sympathy. Some even described positive personality changes due to their caregiving role, stating that they could not imagine going back to who they were before having to care for a child with CP. A father of a 10-year-old boy with diplegia shared,

*My personality changed after having my child (child with CP). I am showing more sympathy.*

(F04)

Ultimately, their beliefs in these Islamic values translated to a feeling of ease and a perception that any happiness they experience in life is because of their child’s condition. For instance, two parents stated,

*My child is the key to my happiness in this life and for Jannah (Heaven).*

M10

*There are many complex things in my life as a single mom for a child with a disability … like my psychological and financial states that suddenly improve because of my child. I didn’t notice them before, but now I feel it because of Allah then my child… before I couldn’t imagine that I would buy a car, but suddenly I bought one.*

M12

#### 3.2.2. Theme 2: The Value of Family and External Support

While all parents discussed the importance of nuclear and extended family members interacting and providing support, the level of perceived family support varied. Those parents with less perceived family support attributed their feelings of loneliness and isolation to the lack of support and understanding from spouses, other children, and extended family. For example, a mother of a 9-year-old boy with diplegia CP stated:

*I wish if my husband and my children could help me more…I wish my husband take care of my son’s appointment. My son does not like to go with his father because he just handles him to a therapist and does not sit with him in physiotherapy sessions… also, we don’t go to my family in-laws because they don’t ask about my son or let him feel welcoming or accepted.*

(M10)

On the other hand, those with positive family support described how having a child with CP and the consequent need to collaborate with their spouses to ensure adequate provision of resources and support had strengthened their family bonds and marital relationship, increasing their cohesion as a family unit. These parents reported that the nuclear family support was a cornerstone in their lives, aiding them in managing daily challenges and contributing significantly to their personal growth and resilience. A mother of a 3-year-old girl with quadriplegia CP shared:

*“Thank Allah, we have increased in intimacy….my husband and I have become a united force. We tirelessly seek the best options for her, aiming to admit her to top-notch facilities and ensure she has all the necessary equipment. Overall, we’ve become diligent in our quest to provide for her.”*

(M05)

Across all interviews, parents appreciated the help provided by extended family. Still, they shared that the involvement of extended family members was somewhat limited to providing emotional support, including kindness shown towards their child with CP. Several parents described a lack of additional tangible social support from their extended family, including practical assistance, respite, and financial aid:

*My brothers and in-laws ask about my child and pray for him… I would be happier if my sister-in-law could take my son and watch him for a short time, as we live close by, to allow me to relax and have some personal time.*

(M04)

Parents further discussed the importance of supportive structures outside their family unit, including their workplace, wider community, and government, noting the varied impact of these external supports on their caregiving role. One father with a supportive community network stated:

*As a community, we are indeed very cooperative, honestly. We have changed a lot; we are not like before… Now, people have come to like individuals like this (children with CP). They don’t leave them; they help them. For example, if we go to parks, people give my daughter a gift, and there are no looks or anything.*

(F01)

In contrast, some parents received little support from their wider community, experienced discrimination, and felt isolated, sad, and disappointed when their child with CP could not join other children for activities in the community or was treated differently by others. These parents described restricting their participation in social activities and interaction with others. For example, one mother shared how she was not comfortable with the comments from community members during social gatherings about her child’s condition and closed the door to comments by saying Allah created her differently because Allah loves her more. Conversely, another mother described how she used discriminatory experiences as a teachable moment to teach her son about resilience instead of retreating socially:

*My son was playing, and some children came to play with him in the park. But they said: You are limping. He came to me and said: Mom, I’m not limping. I told him, ‘Sweetie, you’re not limping, but your leg just needs a little strengthening, and you need to continue to work on your exercise plans.*

(M10)

While parents shared that they received financial support for childcare from the government, such as child benefits, it was often inadequate to meet their needs. Several parents also described difficulty accessing this support. One parent said:

*Since my son’s diagnosis, I have received little guidance on our (parents and the child) rights or how to navigate them. Most of what I have learned has come through my own research or the shared experiences of other mothers*.

(M03)

#### 3.2.3. Theme 3: The Quality of Educational and Healthcare Services

A common theme running through the parents’ accounts was that many aspects of seeking and receiving help were perceived as challenging in the context of education and health services. Parents wanted their children with CP to be independent and have access to education, like other children without disabilities. However, they highlighted experiencing significant challenges in the accessibility, quality, and availability of appropriate educational services for children with CP and their families. For instance, although parents could enroll their children with CP in public schools, the support infrastructure was limited. One father of an 8-year-old girl shared that his wife needs to accompany their daughter on the school bus every morning to help her transition to school and assist her by providing physical support during classes. Then, his wife and their daughter come home from school at noon, and the wife needs to handle household chores. This participant also spoke about advocating for his child’s right to health services, equipment, and housing, as there were no clear guidelines for him on how to access resources to help his daughter.

While school administrators emphasize the importance of accessible, child-friendly, and inclusive school spaces for students with disabilities, many parents of children with CP express that the school’s accessibility standards do not adequately meet the specific needs of their children. One of the mothers of a young girl with quadriplegia CP shared:

*While attending my daughter’s school, I observed a lack of accessibility features. This is particularly concerning as accessibility is important for all students, including those with disabilities. While the school has attempted to install accessible toilets for children with special needs, these facilities unfortunately do not meet my daughter’s specific needs*.

(M11)

Relative to healthcare, parents described feelings of frustration with the healthcare system. Several parents mentioned waiting for long periods before receiving a proper diagnosis and that upon receiving the diagnosis, healthcare professionals did not provide sufficient information to help them understand, cope with, and care for their children with CP. A father of a 9-year-old boy with quadriplegia CP stated:

*I wish someone had informed me about the diagnosis sooner. As a premature baby, he had regular check-up appointments every month, yet no one has delivered a definitive diagnosis. This ongoing uncertainty is concerning and frustrating and has led my wife to explore other traditional ways…. we visited an old lady in (village) who specialized in herbal treatments.*

(F09)

Other parents described feelings of neglect from healthcare providers, describing them as lacking adequate knowledge and understanding of the importance of caregiver well-being. These parents felt that healthcare providers or their workplace needed to support caregivers better to ensure that they can continue in their caregiving role successfully. A working mother shared:

*During my son’s PT sessions, I often fall asleep on the couch because I sometimes come directly from work…. I’m present, observing and asking questions, but no one explains why certain exercises are done or which part of the body they target I wish there were educational courses for parents about physical therapy and exercises, and my boss gave me a flexible schedule*.

(M10)

Furthermore, accessing rehabilitation services for children with CP was challenging for many parents who reported needing to travel for appointments in major rehabilitation centers. Some parents also discussed the challenges of long wait times to access rehabilitation services in publicly funded hospitals and outpatient clinics. When they were able to access publicly funded rehabilitation services, many parents, particularly those whose children required intensive therapy, reported receiving a limited number of sessions that were not sufficient to improve their children’s motor abilities. Although private clinics are an option, parents described them as cost-prohibitive and believed they did not always ensure faster access to rehabilitation services. A working father shared:

*I go to a private clinic in Riyadh twice every month (400 kilometres away from their city) with my wife and my child, as many good clinics are in Riyadh, and one session … I really feel embarrassed every time I ask for leave from my supervisor at my job because most of my son’s appointments are during work hours.*

(F07)

#### 3.2.4. Theme 4: Recommendations for Community-Based Programming, Resources, and Policies

This theme captures a pattern of meaning around hope, unmet needs, and visions for a better support system in Saudi Arabia. Parents highlighted the urgent need for accessible and inclusive education. They called for national policies that guarantee the integration of children with disabilities into mainstream classrooms, supported by individualized learning plans tailored to their needs. Parents also stressed the importance of well-equipped school environments, including teacher training, accessible infrastructure, adaptive tools, and access to support staff such as nurses and therapists.

*Due to the heavy curriculum and tightly packed schedule, my son’s educational needs and capabilities are often overlooked. Sometimes, my son would experience a muscle spasm at school, and neither his father nor the teachers knew how to respond, so they would call me.*

(M10)

Additionally, participants highlighted the importance of community awareness and the establishment of inclusive support groups, recreational activities, and community centers:

*When I came to my son’s appointment, I saw the healthy people occupying the parking space that is allotted to disabled people and their families, which can lead us to park in another spot and walk… when my son arrived at the clinic, he felt tired. Accessible parking spaces are essential for families with children with disabilities. It’s important to have clear policies in place to prevent misuse by others in the community.*

(M10)

Another area of support most needed, as articulated by parents for themselves and their children with CP, was financial assistance, such as grants, scholarships, and financial aid, to help cover the costs of specialized care and equipment, particularly as the child’s need for support increases with their growth. Finally, parents suggested that there should be a transition plan for children with CP to help them transition to adulthood. This plan would include job training programs to empower children with CP and foster a stronger sense of community. One father of a 6-year-old boy with diplegia CP said,

*“When everyone-family, community, government works together, we can build a society where all members, regardless of their abilities, can thrive and contribute meaningfully”*

(F08).

Parents emphasized the crucial role the wider community and government can play in fostering a supportive environment for children with CP and their families. Providing necessary community-based resources and programs can help bridge the gap for children with disabilities, ensuring they have equal opportunities to succeed and that their families have access to tools needed to foster a sense of belonging and connection within their communities.

## 4. Discussion

To the best of our knowledge, this study is the first to explore the experiences and needs of Saudi parents of children with CP. We set out to understand the individual, interpersonal, community, and societal factors that influence the caregiving experiences of parents of children with CP in Saudi Arabia.

Overall, the findings reveal a multiplicity of experiences regarding the parenting of children with CP. The complexity of the caregiving journey, the value of family and external support, the challenges within the health and educational systems, and the ongoing search for community and government support describe the daily lives of parents caring for a child with CP. Herein, we situate our key findings within the published literature on parental caregiving for children with CP. Furthermore, we applied the social-ecological framework as a valuable lens to enhance our discussion and provide a thorough contextualization of our findings across the micro-, meso-, exo-, macro-, and chrono-levels of the framework.

At the microlevel, our findings indicate a shift from the traditional view of mothers as the sole caregivers of children with CP in Saudi families. Through our purposive sampling of both fathers and mothers, we observed that caregiving is increasingly shared, with each parent contributing distinct but complementary roles. Our findings underscore the importance of parental role-sharing, which is crucial for parents to form a fully functioning unit that benefits the child with CP. This finding is inconsistent with the existing Saudi, Arab, and Muslim community literature, which often portrays mothers as the sole caregivers [8,16,49,50]. In this context, gendered norms may sideline fathers of children with CP, limiting their involvement and overlooking their emotional needs. As a result, support programs need to actively engage and empower fathers.

A similar shift toward egalitarian caregiving roles, with an increasing number of fathers taking an active role in caregiving duties, has also been observed in Western contexts such as Australia and America [18,19]. This shift in Western contexts is supported by policies promoting paid leave and recognizing the importance of father involvement in child development [51,52,53]. For instance, some Western countries offer policies such as paid leave or unemployment benefits to support parents in being full-time caregivers, which could be applicable in Saudi Arabia [52]. By acknowledging these distinct gender roles and recognizing the unique contributions of both parents, we can ensure that support services are designed to meet the needs of both mothers and fathers, ultimately enhancing the well-being of the entire family [54]. Only one participant was identified as “separated”, highlighting an underrepresented group. Single-parent households often face greater caregiving and financial burdens [55]. Consequently, future research may explore how single parents of children with CP navigate caregiving roles, especially in the context of persistent gender roles (e.g., Saudi Arabia), to identify gaps in support and inform more inclusive policies.

Our study revealed the multifaceted support needs of Saudi parents of children with CP, identifying the need for four key types of support: emotional support, focusing on improving parents’ psychological well-being; physical support, such as assistance with daily tasks; material/instrumental support, including financial assistance and support with necessary tasks; and informational support, aimed at enhancing knowledge about cerebral palsy. This finding aligns with the work by Kyzar et al. [56], who categorize support similarly as emotional, physical, material/instrumental, and informational. This indicates that parents remain an essential but often invisible part of the Saudi caregiving system. Without adequate support, they are at risk of experiencing social, financial, academic, physical, and mental health challenges, which can profoundly affect their and likely their loved one’s quality of life.

Interestingly, most Saudi parents fulfill their emotional needs through their Islamic spirituality. However, some parents expressed a need for emotional support from nuclear family members (microlevel), as well as extended family and healthcare professionals (mesolevel), particularly during the initial stages of parenting. Physical support needs, such as assistance with daily care tasks (e.g., dressing, lifting, teaching), were predominantly reported by Saudi mothers. The need for assistance in finding financial resources and transportation was mostly reported by fathers and often required support from governmental organizations (exolevel). Consistent with previous research [10], this study found that informational support needs center on the child’s clinical condition, particularly early diagnosis and treatment. Notably, the current study highlighted the importance of supporting parents’ own well-being, a finding not commonly reported in existing Saudi literature. The focus of previous studies in Saudi Arabia has primarily been on parents’ knowledge and attitudes toward the CP condition, as well as societal perceptions of disability [3,49].

Our findings indicate that Islamic spirituality significantly influences the lives of Saudi parents. Spirituality consists of personal beliefs, values, and meaning and religion is deeply rooted in institutional practices and beliefs [7]. While the social-ecological framework encompasses beliefs and culture within the macrosystem, we argue that the personal expression of Islamic spirituality, such as reliance on Tawakkul (trust and reliance on Allah) and Sabr (patience), may not always be articulated in the applied interpretations of the framework [31]. In the stories shared by our participants, these aspects were intertwined and held great importance in their family life. This may be because Saudi Arabia is a deeply religious country with ancient Islamic roots and sees religion permeating cultural identities and values, including attitudes toward disability [23]. Future research needs to explore the role of Islamic spirituality in greater depth within the social-ecological framework. Additionally, there is a need to examine how these spiritual beliefs influence behaviors and decisions during challenging situations. For example, while research in the Islamic Republic of Pakistan suggests that religious beliefs can lead to the rejection of treatment or early intervention services [57], most Saudi parents in our study actively sought medical care. However, participants facing delays and dissatisfaction with the healthcare system did turn to seek traditional or herbal medicine. This variation highlights how caregiving decisions are shaped not only by religious and cultural values but also by context-specific factors such as service accessibility and national discourses around disability.

The recent healthcare changes in Saudi Arabia include Vision 2030 [58], which focuses on improving public health and care services by optimizing the experience and satisfaction of beneficiaries, particularly families of children with disabilities. However, all our participants recalled the barriers faced when seeking appropriate care for their children with CP. These barriers included delays in obtaining an accurate diagnosis, difficulty accessing specialized medical facilities, poor communication with healthcare staff, and limited treatment sessions for rehabilitation therapies. These barriers not only affect the physical well-being of parents but also contribute to the well-being of children with CP. One potential solution to address these barriers is applying a family-centered care approach within the healthcare system. Kokorelias and colleagues [59] identified key elements of family-centered care. These include collaboration between families and providers, consideration of the family’s context, ensuring flexible policies and procedures, and providing education to individuals, families, and support providers. The authors stated that these elements of family-centered care models could be applied to all populations. Family-centered care models have shown promise in Western contexts [59,60]. Therefore, to provide effective family-centered care in Saudi Arabia, healthcare providers must be sensitive to traditional gender roles and Islamic values. For example, Saudi support providers can encourage families of children with disabilities by sharing stories of other families who have successfully used spirituality to cope [61]. This includes respecting the family structure while ensuring opportunities for both parents to participate in decision-making and integrating religious beliefs into care plans.

Saudi Arabia is moving forward in improving education for children with disabilities, and public primary schools are required to accept all children unconditionally. However, in reality, very few children with CP attend school [62]. Our participants highlighted several barriers facing children with CP in education systems, including physical inaccessibility, lack of specialized resources, and inadequately trained staff. These challenges impact both academic progress and social integration. Moreover, parents often find themselves providing additional care for their children when they are not in school. There are numerous implications for this additional caregiving responsibility, including increased psychological stress, financial strain due to lost work opportunities, and reduced time for self-care or supporting other family members. This study’s findings resonate with global research on inclusive education, reinforcing the urgent need for policy reforms and increased investment in inclusive education training for teachers in Saudi Arabia [63].

Our findings at the societal level revealed that many parents required more robust support systems, including increased financial assistance, accessible public facilities and parking spaces, and community education. The findings also highlighted the presence of stigma and discrimination within some communities, which can significantly impact the well-being and quality of life of parents of children with CP. This aligns with Alwhaibi’s study, which found that Saudi parents of children with CP experience a lower quality of life in social and economic domains compared to parents of children without disabilities [14]. Addressing these challenges requires a multifaceted approach that includes raising awareness, promoting inclusivity, and combating stigma. For example, a knowledge mobilization campaign in Canada aimed to raise awareness about the need for caregiver-friendly workplaces and provide workers and employers with access to relevant resources. This initiative and other public awareness campaigns demonstrate the importance of such efforts in reducing stigma and improving knowledge about neurological conditions [64].

As children with CP transition to adulthood, the needs and priorities of their parents also evolve within the chronolevel. Our participants suggested a transition plan to help children with CP transition to adulthood. This plan would include job training programs to empower children with CP and foster a stronger sense of community. A recent review [65] examined the current state of transitional care for individuals with CP. The review showed that the transition from childhood to adulthood for patients with CP is associated with poor outcomes, including housing instability, unemployment, difficulty forming relationships, increased hospital admissions, and decreased use of rehabilitation services. These challenges—unemployment, relationship difficulties, and limited access to services—are precisely the areas our participants identified as key focus points for the proposed transition plan.

Collectively, our findings support the interactions across the social-ecological framework levels. For example, evolving cultural and social values (macrolevel) shape institutional and organizational support (exolevel), influencing workplace dynamics (mesolevel) and, ultimately, caregiving roles within families (microlevel). These findings underscore the need to examine systemic support for parental caregiving holistically rather than in isolation.

### 4.1. Strengths and Limitations

To the best of our knowledge, this is the first qualitative study specifically focused on exploring the experiences and support needs of Saudi parents of children with CP through individual interviews. The use of a social-ecological framework provided a comprehensive lens to identify the multilevel factors that may influence the caregiving experience. Additionally, equal representation of mothers and fathers was achieved through purposive sampling, which may not be reflective of a real-world/natural setting for most caregivers of children with CP. However, some limitations need to be considered when interpreting our results. The findings were translated from Arabic to English, which may have resulted in some loss of meaning. However, the initial analysis was carried out in Arabic, and the second author assessed the translation process. Additionally, Arabic words accompanied by English explanations were used where nuance was difficult to convey. Another limitation is that the interviewer’s gender (female) may have influenced fathers’ responses, despite efforts to build rapport. To address this limitation, we included a male researcher (AS) on the analysis team. The views of 12 participants residing in Qassim province may have limited transferability beyond this context. This homogeneity limits the transferability of findings to parents from diverse socioeconomic backgrounds or those living in other regions of the country. Finally, this study did not capture household size, which could have provided insight into the living arrangements and other shared caregiving responsibilities of parents of children with CP.

### 4.2. Practical Implications

The findings of this study offer actionable insights that can directly inform healthcare and social support reforms under Saudi Arabia’s Vision 2030 [58]. As the nation strives to improve the quality of life for families, empower individuals with disabilities, and foster inclusive healthcare systems, our findings underscore the pressing need for comprehensive parent training programs. These programs should equip parents and caregivers with practical skills in home-based rehabilitation, stress management, and navigating healthcare services [66,67,68,69]. Additionally, the Ministry of Education should implement inclusive school policies that facilitate the enrollment and support of children with CP, including the training of teachers [70] and the provision of accessible infrastructure [71]. Also, raising public awareness about CP is crucial. For instance, awareness campaigns can dispel myths, reduce stigma, and encourage community engagement [64], which in turn can lead to increased support for families affected by CP. Ultimately, establishing community-based support networks and peer groups can help alleviate caregiver isolation and foster social cohesion. These implications support Vision 2030’s goals of creating an inclusive, resilient, and family-centered landscape [58].

## 5. Conclusions

This study provided novel insights into the multifaceted nature of the experiences of Saudi parents of children with CP. Our findings highlighted the active involvement of both mothers and fathers in caregiving, moving beyond traditional assumptions of maternal demonstration. This study revealed limited access to adequate healthcare and educational systems tailored to the needs of Saudi children with CP and their parents. Consequently, we emphasize the importance of considering the unique cultural and social contexts—including Islamic values and gender roles—when developing effective support systems for Saudi families. By addressing the diverse needs and challenges faced by Saudi parents, we can enhance the well-being of both parents and children with CP. Further studies are needed to evaluate the community-based programming, resources, and policies proposed by parents in this study.

## Figures and Tables

**Table 1:** Characteristics of parents and their children with CP.

Variable	*n* (%)
**Parents (caregivers)**	
**Gender**	
Female	6 (50)
Male	6 (50)
**Marital status**	
Married	11 (91.7)
Separated	1 (8.3)
**Level of education**	
High school or less	7 (58.3)
Bachelor’s degree	5 (8.3)
**Monthly family income (Saudi riyals (SAR))**	
<6000	6 (50)
≥6000	6 (50)
**Children with CP (care-recipients)**	
**Gender**	
Female	4 (33)
Male	8 (67)
**CP type**	
Hemiplegia	1 (8.3)
Diplegia	7 (58.3)
Quadriplegia	4 (33.4)
**Motor skill**	
GMFCS ^1^ II	5 (41.67)
GMFCS IV	3 (25)
GMFCS V	4 (33.33)
	**Mean (SD** ^2^**)**
Age of parents, years	45 (11)
Age of children, years	8 (4)

^1^ GMFCS: Gross Motor Function Classification System; ^2^ SD: Standard deviation.

## Data Availability

Data are available by a reasonable request to the corresponding author.
